# At the Moment of Death

**Published:** 1892-05

**Authors:** 


### AT THE MOMENT OF DEATH.

Some years ago, in a small street, the workmen employed in the excavations at Pompeii discovered an empty space of an unusual form, in which were some skeletons. Before disturbing them they called Signor Florelli, who was fortunately at hand. A singularly happy thought struck him. He had the empty space filled with liquid plaster of Paris and repeated the process in the case of some other openings which presented a similar appearance. As soon as the plaster was hardened the surrounding ashes were removed and displayed the perfect casts of four human bodies. All four are now in the museum there, and a more singular and affecting sight is, perhaps, not to be seen in the world. The plaster was hardened around the ashes so perfectly in shape of what may be termed the mold formed by the fallen ashes round the living bodies that the whole aspect of the dying frame is preserved, even to the minutest details, except that here and there the bones of the skeleton within are partially uncovered. Egyptian mummies are bare, black and hideous. and arranged in an artificial posture for their burial, while in the exhumed Pompeiians we see human beings in the very act of dying.

One of them is the body of a woman, close to whom were found a large number of coins, two silver vases, some keys and some jewels, which she was carrying with her when the falling ashes arrested her flight. It is easy to trace her head-dress and the material of her clothing, and on one of her fingers are two silver rings. Her hands were so clasped in agony that the nails had pierced the flesh. With the exception of her legs, the whole body is swollen and contracted. It is plain that she strove violently in her dying struggle. Her attitude is that of the last agony and not that of death. Behind her lay another woman and a girl, evidently of humble rank.

The elder of the two, possibly the mother, has an iron ring on one of her fingers. The signs of a dying struggle are evident, but the death seems to have been easier than in the case of the victim last described. Close to her lies the girl, almost a child in age. The details of her dress are preserved with a startling faithfulness. One can see the material and stitching of her frock, the unmended rents in her long sleeves, her dress over her head, to ward off the torrent of ashes, and falling headlong on her face had rested her head on one of her arms, and so died, apparently without a struggle.

The fourth body is that of a large and powerful man who had sat down to die with his arms and legs straight and fixed. His dress is completely preserved, his trousers are close, his sandals are laced to the feet, with nails in their soles. On one finger is an iron ring; his mouth is open, and shows that he had lost some of his teeth; his nose and cheeks are strongly marked; the eyes and hair have disappeared, but the moustache remains. The whole sight is tragic to the last degree. After the lapse of eighteen centuries the terrible death seems to be enacting itself before us with all its appalling sufferings.

